# Use of high hydrostatic pressure to inactivate natural contaminating microorganisms and inoculated *E*. *coli* O157:H7 on *Hermetia illucens* larvae

**DOI:** 10.1371/journal.pone.0194477

**Published:** 2018-03-22

**Authors:** Mahboobeh Kashiri, Cuauhtemoc Marin, Raquel Garzón, Cristina M. Rosell, Dolores Rodrigo, Antonio Martínez

**Affiliations:** 1 Department of Food Science and Technology, University of Agricultural Sciences and Natural Resources, Gorgan, Iran; 2 Instituto de Agroquímica y Tecnología de Alimentos (IATA-CSIC), Catedrático Agustín Escardino 7, Paterna, Valencia, Spain; Universita degli Studi della Basilicata, ITALY

## Abstract

A chemical and microbiological characterization on *Hermetia illucens* larvae was carried out as well as an inactivation study of natural contaminating microorganisms and inoculated *E*. *coli* O157:H7 in black soldier larvae by using High Hydrostatic Pressure (250 to 400 MPa, for 1.5 to 15 min). *Hermetia illucens* was mainly composed of proteins (46.49%, d.m.) followed by fat (37.88%, d.m.). Larvae had a high contamination load of Total Aerobic Mesophilic bacteria (AMB) (1.58x10^7^ cfu/g) and Enterobacteriaceae (1.15x10^6^cfu/g). The presence of pathogenic microorganism varied: no *Listeria* spp. were found, but *Salmonella* (1.15x10^6^ cfu/g) and *E*. *coli* (7.08x10^5^ cfu/g) were detected in the larvae extract. High Hydrostatic Pressure (HHP) was effective against natural contaminating yeasts and molds producing more than 5 log cycle reductions at 400 MPa for any of the times considered (2.5 to 7 min), but a low reduction of total microbial load was achieved. The inactivation level of larvae inoculated with *E*. *coli* O157:H7 varied. At 400 MPa for 7 min more than 5 log cycle reductions were achieved. Among the three inactivation models studied, the one that best described the inactivation pattern of the cells, according to the Akaike index, was the Biphasic model.

## Introduction

Trends predict a steady increase in population, reaching nine billion people in 2050 and forcing an increase in production of food and feed. This may affect agricultural ecosystems and the consequence could be a shortage of land for cultivation, water, forests, fisheries, and biodiversity resources, as well as nutrients and nonrenewable energy [1]. Insects, of which there are about 2,000 edible species, have always been part of the human diet [1, 2]. Consequently, the use of edible insects could be a sustainable protein supply, either for direct human consumption or indirectly in new foods made from insect protein; and has recently been promoted by the FAO as a protein source in feedstock mixture.,. In a study carried out by [3] on quality of proteins from edible indigenous insect food of Latin America and Asia, authors concluded that those insects have a good nutritional value and are high in protein with all essential amino acids.

Edible insects are usually cooked in salted water, dried on large surfaces, or slightly roasted before consumption [4, 5]. The current insect processing industry has begun offering dried products, using conventional or freeze-drying techniques [6]. However, sanitary authorities in Europe advise that these insects should be suitably heated before consumption [7]. Raw insects are characterized by elevated bacterial and fungal counts [7]. Those microorganisms often contaminate external parts of insects as shell as their intestinal tract, and neither conventional methods (degutting, boiling, sun-drying or roasting) nor modern freeze-drying techniques seem to be fully effective against all microorganisms, they can remain contaminating the product and when rehydrated, many could return to vegetative stadia. [8].

Processing must ensure the safety of the product while preserving its nutritional value. Various preservation methods (e.g. using UV, light, pH, high hydrostatic pressure) could be applied to remove possible pathogenic microorganisms. *E*. *coli* is a known foodborne pathogen that frequently causes foodborne illness outbreaks [9] *Enterobacteriaceae*, *Staphylococcaceae*, yeasts and molds, and Bacilli have been found in the feed substrate of edible insects sold as pet feed in Germany [6], considering those finding, pathogenic microorganisms as *E*. *coli* could be found in the material used in rearing insects.

Among the possible insects to be used as protein sources, the larvae of the black soldier fly (*Hermetia illucens)* is a very efficient organism that can be used in the management of organic waste [10], and in animal feed [11]. However, one of the main concerns of the feeding system is the hygiene of the pre-pupae and of the compost that is produced [12].

High hydrostatic pressure (HHP) is one of the most popular non-thermal preservation technologies. The effect of this treatment has been demonstrated to be capable of inactivating *E*. *coli* in beef meat [13], poultry meat [14], vegetables [15], ovine milk [16], fruits [17], seafood [9], between other foods, but according to [18], up to today it has not been used for the decontamination of edible insects.

Some studies to assess the impact of cold plasma, high hydrostatic pressure, and thermal treatments have been carried out on the microbial surface of *Tenebrio molitor* (meal worm) larvae [18]. Results indicated that high hydrostatic pressure at 600 MPa and thermal treatments in a water bath at 90°C induced the highest reduction of the total count of microorganisms contaminating *Tenebrio molitor*. Consequently, high hydrostatic pressure could be a promising technology to reduce the microbial load of insects before consumption of them as food and feed.

In the present work, black soldier larvae were chemically and microbiologically characterized, and high hydrostatic pressure technology was used to inactivate natural contaminating microorganisms and inoculated *E*. *coli* O157:H7. Inactivation data were fitted to different mathematical models; those models are useful in an exposure assessment when a quantitative risk assessment is needed.

## Material and methods

### Larvae

Larvae were supplied by BioFlyTech, S.L. (Alicante, Spain). Black soldier fly larvae were reared on barley chaff and harvested in pre-pupae stage. After harvesting, they were frozen at -20°C for 1 h to kill them and then dried at 80°C for 2 hours. Dried black soldier fly larvae were ground using an IKA M20 laboratory mill (IKA-Werke GmbH & Co. KG, Staufen, Germany) and the resulting powder was used for microbiological and chemical characterization.

### Chemical characterization

Fat and crude protein contents of larvae were determined in duplicate by the AOAC methods 960,39 and 981,10, respectively [19] by using a 2055 Soxtec (Foss Tecator, Sweden) and 2200 Kjeltec auto distillation unit (Foss Tecator, Sweden), respectively.

Proteins content was determined in the residue after precipitating the proteins with trichloroacetic acid and then evaluated as described in the above mentioned method Moisture was determined by infrared method at a constant temperature of 130°C for 30 seconds, using a Kern DBS 60–3 (Kern & Sohn GmbH, Germany), in duplicate. Ash content was measured following the method specified by ISO 2171:2010 for cereals, pulses and by-products. Nitrogen-free extract (NFE) was determined by difference (NFE = 100%–(protein + crude fat + ash + crude fiber + moisture). Fat, protein, ash and NFE were expressed in dry matter.

### Microbiological characterization

For each determination, 10.0 g ± 0.1 g sample with 90 ml peptone water (Scharlab, S.L, Barcelona, Spain) was homogenized for 3 min in a Stomacher (Scharlab, S.L, Barcelona, Spain). Then the following methods, UNE-EN ISO 11290–1, UNE-EN ISO 6579, UNE-EN ISO 4833, UNE-EN ISO-21528-2, UNE-EN ISO 16649–1, UNE-EN ISO 7954, were used to determine *Listeria* spp., *Salmonella* spp., aerobic mesophilic bacteria, Enterobacteriacea, *E*. *coli* spp., and yeasts and molds, respectively.

### Culture preparation for inactivation studies

*E*. *coli* CECT 910 (*E*. *coli* O157:H7*)* was supplied by the Spanish Type Culture Collection and used as inoculum of larvae. For rehydration of the lyophilized strain, it was transferred to 10 ml of Tryptic Soy Broth (TSB) (Scharlab Chemie S. A., Barcelona, Spain). After 30 min, 5 ml of culture was inoculated in 200 ml of TSB and incubated at 37°C with constant agitation at 200 rpm for 6 h. Then, 40 ml of the culture was transferred into 400 ml of TSB and incubated for 12 h at 37°C with constant agitation at 200 rpm. After incubation, cells were centrifuged twice at 4000 × g for 15 min at 4°C and re-suspended in 20 ml of TSB. Then, cells were placed into 2 ml sterile plastic cryogenic vials containing TSB supplemented with 20% glycerol. The 2 ml samples were immediately stored at –80°C until they were needed. The approximate concentration of each sample was 3 × 10^7^ CFU ml^–1^

### Sample preparation for inactivation studies

In these studies, uninoculated and inoculated larvae were used. In the case of inoculated larvae, 5 g of larvae suspended in 6 ml of peptone water (0.1%) were heated at 121°C for 15 min to allow sterilization, and then stored at 4°C until use. Stored samples were aseptically transferred to polyethylene bags, and 5 ml of sterilized peptone water was added. For inactivation studies, 1 ml of microorganism culture from cryogenic vials was transferred to 9 ml of peptone water, and 1 ml of this cell suspension was transferred to each bag.

For uninoculated samples, 5 g of larvae suspended in 11 ml of sterilized peptone water (0.1%) were placed in polyethylene bags.

Finally, bags containing uninoculated or inoculated larvae were vacuum sealed and placed in the high hydrostatic pressure chamber for treatment.

### High hydrostatic pressure treatment

High hydrostatic pressure treatments were performed in a pilot-scale unit (High-Pressure Food Processor, EPSI NV, Belgium) with a vessel operating pressure of 2.35 litres and a maximum treatment pressure of 600 MPa. The pressure transmitting fluid was a mixture of water and ethylene glycol (70:30, v:v). The samples were pressurized at 250, 350, and 400 MPa for 0 to 15 min. All the treatments were applied in duplicate, (two repetitions), with at least two replicates per treatment and two bags per replica. After completing the treatment, the samples were removed from the vessel and immediately transferred to an ice-water bath and stored under refrigeration (3 ± 1°C) until needed for analysis. In all cases, an unpressurized inoculated bag was used per repetition with the subsequent replications as a control of the initial microbiological load.

### Enumeration of microorganisms

Serial decimal dilutions of the treated samples and the controls were performed in 0.1% sterile peptone water (Scharlab Chemie S. A., Barcelona, Spain). The enumeration medium used for *E*. *coli* viable cells was Tryptic Soy Agar (TSA) (Scharlab Chemie S. A., Barcelona, Spain), for total aerobic microorganisms it was Plate Count Agar (PCA) (Scharlab Chemie S. A., Barcelona, Spain), and for yeasts and molds it was Potato Dextrose Agar (PDA) supplemented with 10% of tartaric acid (Scharlab Chemie S. A., Barcelona, Spain). The selected dilutions were incubated at 37°C for 24 h and at 30°Cfor 48 h for *E*. *coli* and total aerobic microorganisms, respectively, and at 25°C for 5–7 days in the case of yeasts and molds. The reduction of viable cells was expressed as the decimal logarithm of the counts.

### Mathematical models

GInaFiT software [20] was used to fit survival data of the *E coli* O157:H7 treated by high hydrostatic pressure, to different mathematical models. The mathematical models contained in the GInaFiT software used in this study were: the Weibull model (Eq 1), [21, 20], the Cerf model with shoulder (Biphasic model) (Eq 2) [22], and the Log-linear model (Eq 3) [23].

$$Log_{10}\left(N\right)=Log_{10}\left(N(0)\right)-{\left(t/\delta \right)}^{p}$$ Eq 1

where *N* (CFU / mL) represents the final concentration of cells, *N (0)* (CFU / mL) is the initial concentration of cells; *t* is the time (min); *δ* is the scale parameter; *p* is the shape parameter, which corresponds to a concave upward curve if *p* < 1, a downward convex curve if *p* > 1, and if *p* = 1 it describes a linear behavior.

$$Log_{10}(N)=Log_{10}(N(o))+Log_{10}(f\cdot e^{-k_{\max1}t}+(1-f)\cdot e^{k_{\max2}t})$$ Eq 2

where *N* (CFU/mL) represents the final concentration of cells; *N*(*0*) (CFU/mL) is the initial concentration of cells; *f* is the fraction of the initial population considered as the bigger subpopulation, (1–*f*) is the fraction of the initial population considered as the smaller subpopulation (which is more heat resistant than the previous subset); *k*_*max1*_ and *k*_*max2*_ (1/time unit) are the rates of inactivation for the two subpopulations, respectively; *t* is the time (min)

$$N(t)=(N(0)-N_{res})\cdot e^{-k_{\max}t}\cdot \left(\frac{e^{k_{\max}S_{1}}}{1+(e^{k_{\max}S_{1}}-1)\cdot e^{-k_{\max}t}}\right)+N_{res}$$ Eq 3

where *N* (CFU / mL) represents the final concentration of cells; *N* (*0*) (CFU/mL) is the initial concentration of cells; *N*_*res*_ is the concentration of residual cells (CFU/mL); *k*_*max*_ is the specific inactivation rate (1/unit time); S_1_ is the parameter representing the time needed to appear significant inactivation (shoulder) (time units); t is the time (min).

### Model comparison

The criteria and parameters used to compare the goodness of fit of the models were: Adjusted coefficient of multiple determinations (Adj*R*^*2*^), Estimated Standard Deviation of the Regression Error Term (*RMSE*), and the Akaike Information Criteria for Model Selection [24].

The Akaike Information Criterion *(AIC)* is also a way of selecting a model from a set of models. Two measures associated with the *AIC* can be used to compare models: the delta *AIC* and Akaike weights. These are easy to compute, as calculations remain the same regardless of whether the *AIC* or *AICc* is used, and they also have the advantage of being easy to interpret. The delta *AIC* (*Δi*), is a measure of each model relative to the best model, and is calculated as:

Δi=AICi−minAIC Eq 4

where *AICi* is the *AIC* value for model *i*, and min *AIC* is the *AIC* value of the best candidate model. Models having *Δi* < 2 suggest substantial evidence for the model, values between 3 and 7 indicate that the model has considerably less support, and *Δi* > 10 indicates that the model is very unlikely [25].

### Statistical analysis

The experimental design and the data analysis were performed using the Statgraphics® Centurion XV software (Statpoint Technologies, Inc., USA).

## Results and discussion

### Chemical characterization

The moisture content of the larvae was rather low (4.79±0.34) because they were dried to extend their stability during storage. Proteins represent the main component of the nutrient composition of insects, between 20% and 77% based on dry matter [26]. Protein content of the *H*. *illucens* larvae was 46.49% (Table 1). Nevertheless, [27] recently reported that according to amino acid analysis a specific Kp of 4.76 should be applied to this larvae, in which case the protein content would be 35.41. However, for comparison purposes with previously reported protein values the widely accepted protein factor (6.25) was applied. Result obtained was consistent with the results reported for different species of flies (Diptera), which were in a range of 35 to 64% [28, 29]. Lower protein content of the same larvae species but fed with poultry manure (37.88%) [30] and food waste (42%) has been reported [31]. The second main component was total fat (38.63%), whose content was slightly higher than the values reported for other species of Diptera such as *Eristalis* sp. (12%) or *Ephydra hians* (36%) [29], probably owing to feeding variation, as mentioned with regard to protein content. Fiber and ash content were within the ranges described for different diptera, which averages were 13.56% and 10.31%, respectively [26]. No amount of nitrogen free extract was detected, which includes mainly carbohydrates, which agrees with previous information reported for some species of Diptera, where carbohydrates comprised mainly fibers [26]. The amount of protein in *H*. *illucens* larvae was higher than the values reported for pork edible flesh (11.9%), beef (17.7%), chickpea (20.1%), hen egg (12.4%), and soybean seed (38%), expressed as dry base [32]. Some studies have shown that black soldier fly meal can replace at least 25% of the fish meal in a diet [11] or 25% of the fish in a diet [33]. Therefore, the high protein content of the *H*. *illucens* larvae dried powder might become an important protein source.

**Table 1:** Nutritional composition of Hermetia illucens (expressed as dry matter).

Parameter	Composition (g/100 g dm)
Total proteins	46.49±0.09
Total fat	38.63±0.19
Ash	4.69±0.02
NFE	n.d.
Crude fiber	11.39±0.09

n.d. Not detected

### Microbiological characterization

Microbial characterization results are shown in Table 2. High counts can be observed for Total Aerobic Mesophilic bacteria (1.58x10^7^±1.82x10^6^ cfu/g) and Enterobacteria (1.15x10^6^±2.58x10^5^cfu/g). Similar results were reported by other authors [34]. Levels of 10^7^ cfu/g for Total Viable Count and 10^4^−10^6^ cfu/g for Enterobacteriaceae were found in fresh edible insects [18].

**Table 2:** Microbiological characterization in Log CFU/g of dry larvae.

Microorganism	Counts (CFU/g)
Aerobic mesophilic	1.58x10^7^±1.82x10^6^
*E*. *coli*	7.08x10^5^±1.8x10^5^
Enterobacteria	115x10^6^±2.58x10^5^
*Salmonella* spp.	1.15x10^6^±4.86x10^5^
*Listeria* spp.	n.d.
Yeasts and molds	5.81x10^6^±1.72x10^6^

n.d. = Not detected.

Counts for pathogen microorganism varied. Although no *Listeria* spp. were found, *Salmonella* (1.15x10^6^±4.86x10^5^cfu/g) and *E*. *coli* (7.08x10^5^±1.81x10^5^cfu/g) were detected in the larvae extract, consequently preservation treatments would be needed to guarantee food safety in relation to those pathogens.

It is necessary to take into account that *H*. *illucens* larvae can grow in a wide variety of waste material. The larvae used in this study were grown in barley chaff rich in cellulose, probably contaminated with high counts of yeasts and molds as well as spores and pathogenic microorganisms that can contribute to the high counts of Total Aerobic Mesophilic bacteria.

### Inactivation of natural contaminating microorganisms in larvae

**Fig 1:**
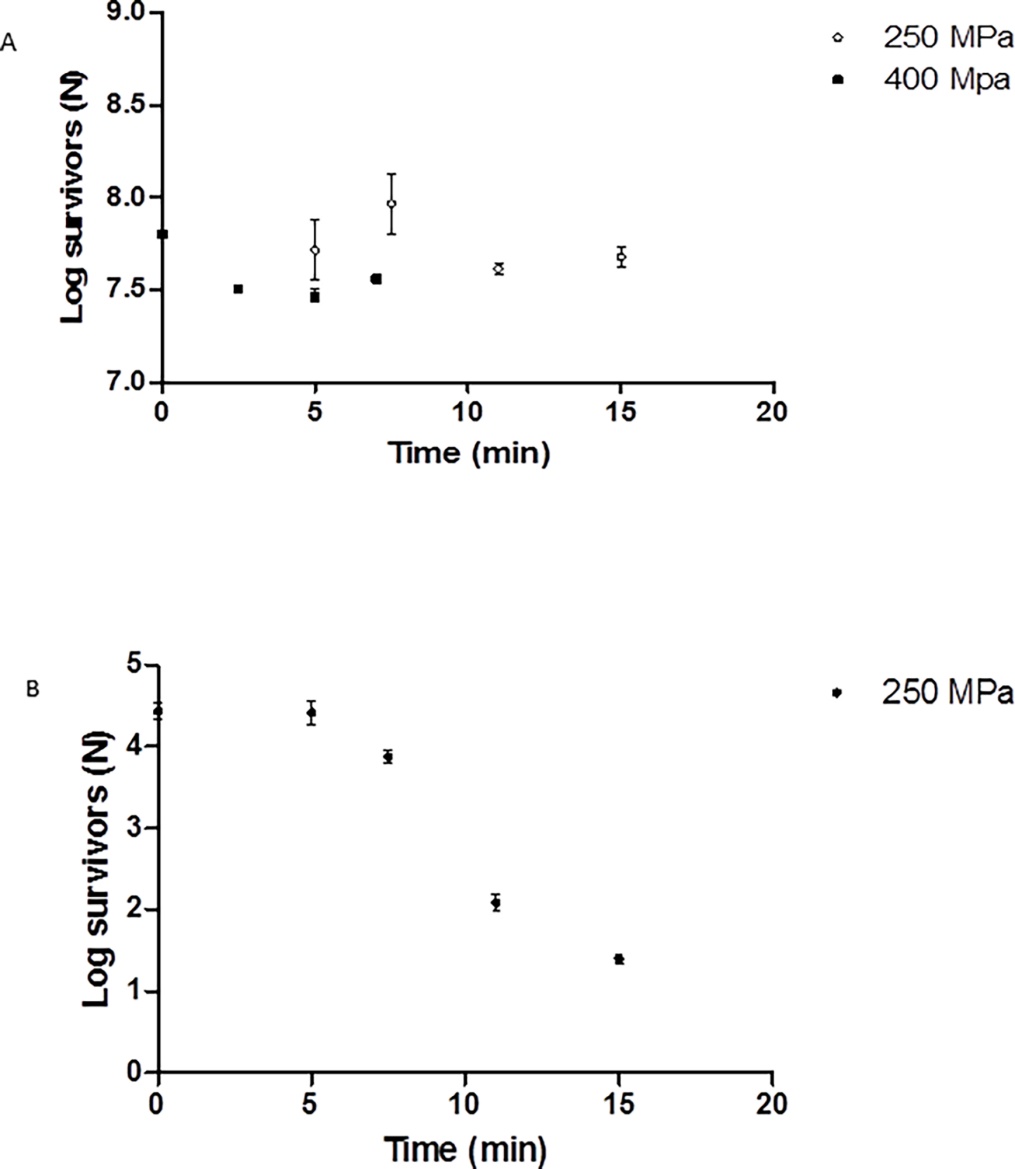
Survival curves for natural contaminating microorganisms (S1 Dataset). (A) total mesophilic aerobes treated at 250 and 400 MPa; (B) yeasts and molds treated at 250. No survivors were observed at 400 MPa for yeast and mold.

The results for inactivation of yeasts and molds and Total Aerobic Mesophilic bacteria in larvae samples treated by using various pressures and times are shown in Fig 1 (S1 Dataset). In general, microbial count decreased with increasing pressure and time of treatment. The logarithm of survivors of yeasts and molds showed a reduction close to 3.03 log cycles obtained by applying a pressure of 250 MPa for 15 min, while at the same conditions, the reduction achieved for Total Aerobic Mesophilic bacteria counts was only about 0.12 log cycles. In the current study, no surviving yeasts and molds were found in the larvae samples after using a pressure of 400 MPa for any of the times considered (2.5 to 7 min), while only 0.35 log reductions were achieved for Total Aerobic Mesophilic microorganisms after 7 min of treatment (Fig 1) (S1 Dataset). Hence, treatment at 400 MPa is capable of effectively controlling the yeasts and molds of larvae. The low effect of high hydrostatic pressure, on Total Aerobic Mesophilic bacteria at the treatment conditions considered in the present work might be due to the presence of microbial spores or the absence of enough water in the vacuum sealed bags, despite of larvae were moisten, as indicated by, [18]. Nevertheless, little information could be found in the literature about inactivation of insect larvae natural flora by HHP. It has been reported that high hydrostatic pressure of 600 MPa for 10 min reduced the Total Viable Count on the surface of mealworms by 3 log cycles [18]. Inactivation of yeast in other foodstuffs has been reported [35]. Those authors indicated that 300–600 MPa for 5 min effectively controlled the occurrence of spoilage yeasts in cheese while in the present work 400 MPa for 2.5 min was enough for totally inactivate mold and yeasts.).

Other treatments as the direct plasma treatment had been used by [18]. Authors concluded that components emitted by the plasma jet had little detectable inactivating effect on the surface microflora of mealworm larvae.

According to [18] the indirect plasma treatment resulted in the best surface decontamination procedure of mealworm larvae comparing all treatments considered in their study (HHP, heat treatment, direct and indirect plasma treatments.

### Inactivation of E. coli O157:H7 inoculated in larvae

**Fig 2:**
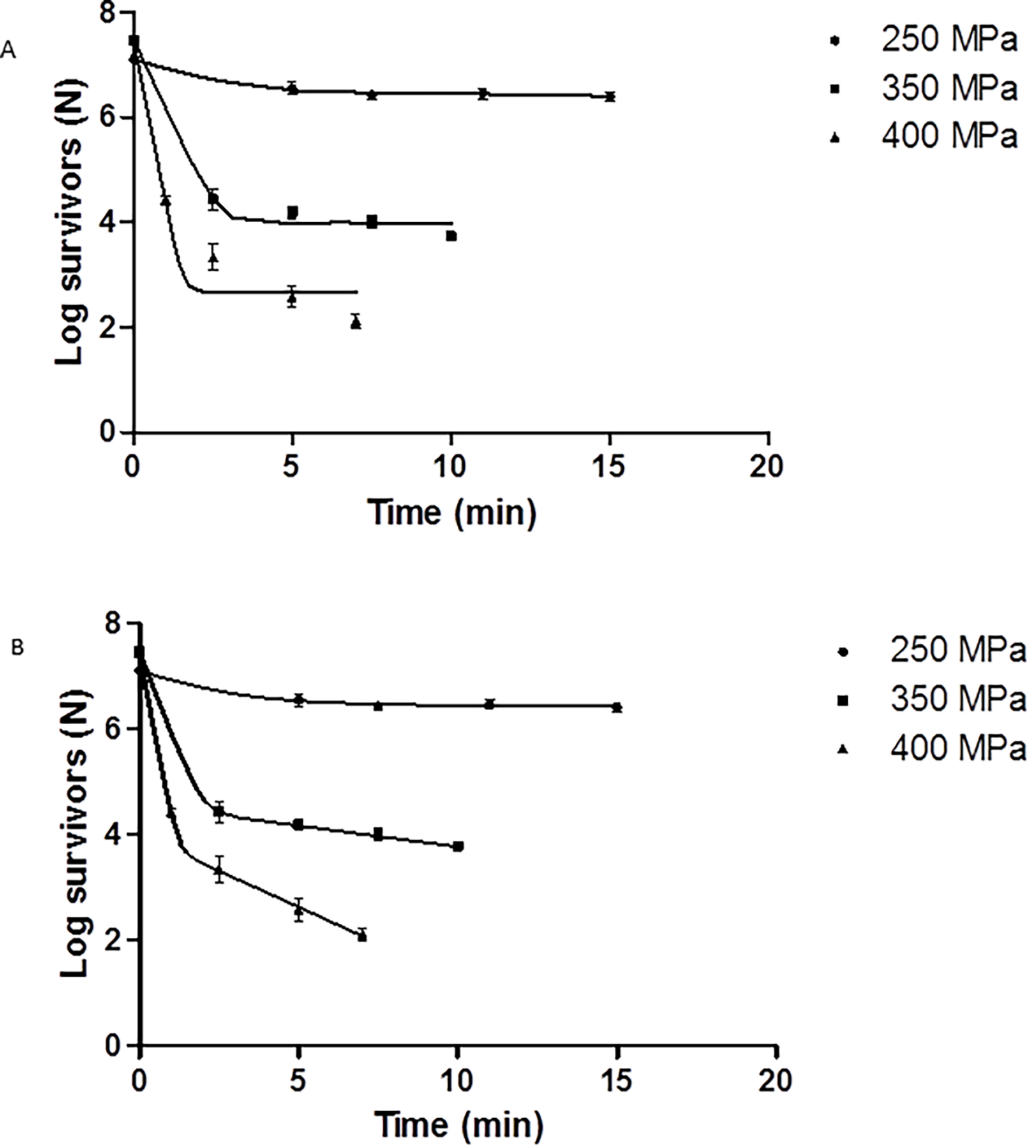
Survival curves for *E*. *coli* O157:H7 and fitted lines (S2 Dataset). (A) for Log-linear model and (B) for Biphasic model on black soldier larvae at 250 MPa, 350 MPa, and 400 MPa.

Inactivation results obtained in the present work for *E*. *coli* O157:H7 inoculated in *Hermetia illucens* larvae can be seen in Fig 2 (S2 Dataset). As can be seen in the Fig 2 (S2 Dataset), the number of decimal reductions on *E*. *coli* survivors increased as the pressure and treatment time were increased achieving a maximum of a 6.56 log-cycle reduction in viable cell numbers of *E*. *coli* O157:H7 in larvae after a treatment at 400 MPa for 7 min. This value, 6.56 log-cycle reduction, is largest than that considered as safe (5 log cycles reduction) for FDA for non-thermal technologies. For other pressure-time combinations applied in this study, a reduction of less than 1.5 log cycles was obtained by applying a pressure of 250 MPa for 5 min. This result was similar to that obtained by [36] when the same bacterium was inoculated in TSBY broth. Those authors [36] achieved reductions of 1.39 and 1.47 CFU/ml after subjecting samples to a treatment of 276 MPa for 5 and 10 min, respectively. Treatment at 350 MPa for 10 min produced a reduction of 3.93 log cycles for *E*. *coli*, and at 400 MPa for 1 min the reduction was 2.78 log_10_ CFU/g. Similar results were reported for *E*. *coli* in minced mild smoked rainbow trout derived from fillet [9], where a reduction of about 2.3 log_10_ units of *E*. *coli* occurred after 1 min with 400 MPa.

The inactivation curves for the pressures studied (250, 300, and 400 MPa) never followed a straight line; thus they could not be analyzed by using a log-linear model such as the Bigelow model (Fig 2). Mathematical models are of paramount interest in quantitative risk assessment. They are generally used in the exposure assessment step of the analysis. Models are also useful in case of changing some environmental or process factors that can affect the behavior of the microbial inactivation, in this way they permit developing new safe processing conditions.

An attempt to model the inactivation of *E*. *coli* O157:H7 on black soldier larvae was carried out. Non-log-linear curves obtained were characterized by a prolonged tail. The curves were fitted to different mathematical models by using GInaFiT [20] to identify the best model for the inactivation curves.

Table 3 shows the parameters for the different models tested. In general, the model parameters suggested that as the pressure increased the resistance of the microorganism decreased. The Weibull model [21] did not fit the inactivation curve at 250 MPa, but it had a good fit for the survival curves at the other pressure levels used in this study (Table 4). The Log-linear with tail model [23] fitted experimental data properly for all the pressures studied, but the one that fitted the experimental data best was the Biphasic model [22] (Table 4). According to these results, the last two models show a good fit as indicated by the low root mean square errors (RMSE) and can be used to describe the inactivation curves of *E*. *coli* O157:H7 in black soldier larvae. Nevertheless, the Akaike Information criterion was applied for model selection. Table 5 shows the values for the Akaike increments (*Δ*_*i*_). Models with *Δi* ≤ 2 have substantial support and should receive consideration when making inferences. Models having *Δi* of about 4 to 7 have considerably less support, while models with *Δi* ≥ 10 have essentially no support, and might be omitted from further consideration. In our study, only the Biphasic model has a *Δi* ≤ 2. This means that this model should receive consideration when making inferences, despite the fact that it has more parameters than the Log-linear with tail model.

**Table 3:** E. *coli* O157:H7 kinetic parameters obtained for each mathematical model used in the study.

Pressure [MPa]	Weibull δ ± σ [min*ml/CFU]	Log-linear with tail *k*_max_ ± σ [CFU/ml*min]	Biphasic	
			*k*_max1_ ± σ [CFU/ml*min]	*k*_max2_ ± σ [CFU/ml*min]
250	NA	0.53±0.19	0.55±0.42	0.003±0.05
350	0.11±0.10	2.95±0.21	3.59±1.18	0.19±0.07
400	0.42±0.34	7.01±0.86	7.23±0.59	0.63±0.11

σ = Standard deviation.

NA = Not available.

**Table 4:** Goodness of fit for the various models used to describe the experimental data (E. coli O157:H7).

Pressure (MPa)	Weibull		Log-linear with tail		Biphasic	
	RMSE^(a)^	Adj*R*^*2*^^(b)^	RMSE^(a)^	Adj*R*^*2*^^(b)^	RMSE^(a)^	Adj*R*^*2*^^(b)^
250	NA	NA	0.140	0.781	0.143	0.764
350	0.321	0.952	0.242	0.971	0.202	0.983
400	0.734	0.861	0.526	0.935	0.313	0.971

^(a)^ Estimated root mean square of the nonlinear regression model.

^(b)^ AdjR^2^, Adjusted coefficient of multiple determination.

NA = Not available.

**Table 5:** Akaike increments (*Δi*) for the various models used to interpret the experimental data (E. *coli* O157:H7).

Model	250 MPa	350 MPa	400 MPa
Log-linear with tail	6.29	29.30	23.7
Biphasic	0	0	0

## Conclusions

According to chemical characterization carried out in this study, black soldier larvae could be used as a protein source for animal feeding as well as to produce human foodstuffs. *Hermetia illucens* larvae has high levels of microbial contamination and some of the contaminating microorganisms are *E*. *coli* and *Salmonella-* The presence of those microorganisms encourage the need of using some control measures if they will be used to be processed as feed or foods. Although the effect of HHP considering the applied conditions of this study had a limited effect on Total Aerobic Mesophilic bacteria, HHP shown its capability in controlling mold and yeast and produce enough log-decimal reductions on *E*. *coli* O157:H7 load ensuring the safety of the larvae for this microorganism. It is necessary more research and data on the impact of high hydrostatic pressure treatments on other pathogenic microorganisms that can contaminate the surface of *Hermetia illucens* larvae in order to develop effective decontamination conditions and ensure the microbial safety of those larvae as food and feed materials. Those studies should be developed by using including *Hermetia illucens* larvae reared on different legally admitted substrates for food and feed.

## Supporting information

S1 Dataset Dataset for natural contamination. (DOCX)

S2 Dataset Dataset for *E coli* O157:H7. (DOCX)
