# Supplementary material for: Use of high hydrostatic pressure to inactivate natural contaminating microorganisms and inoculated E. coli O157:H7 on Hermetia illucens larvae
Source: PLoS One. 2018 Mar 22;13(3):e0194477. doi: 10.1371/journal.pone.0194477 (PMC5864016; doi:10.1371/journal.pone.0194477)
Supplement: S1 Dataset — (DOCX) [file pone.0194477.s001.docx]

**S1_Data set for natural contamination**

Inactivation natural Total Aerobic Mesophylic Bacteria data set

Data for two repetitions per treatment time (log cfu/mL)

|  | 250 MPa | | 400 MPa | |
| --- | --- | --- | --- | --- |
| Treatment time (min) | Repetition1 | Repetition 2 | Repetition1 | Repetition 2 |
| 0,0 | 7,822495 | 7,781755 |  |  |
| 0,0 |  |  |  |  |
| 5,0 | 7,552060 | 7,876795 |  |  |
| 5,0 |  |  |  |  |
| 7,5 | 7,801061 | 8,130012 |  |  |
| 7,5 |  |  |  |  |
| 11,0 | 7,586024 | 7,643206 |  |  |
| 11,0 |  |  |  |  |
| 15,0 | 7,729974 | 7,625312 |  |  |
| 15,0 |  |  |  |  |
| 0,0 |  |  | 7,822495 | 7,781755 |
| 0,0 |  |  |  |  |
| 2,5 |  |  | 7,513217 | 7,492760 |
| 2,5 |  |  |  |  |
| 5,0 |  |  | 7,501744 | 7,434569 |
| 5,0 |  |  |  |  |
| 7,0 |  |  | 7,587711 | 7,537189 |
| 7,0 |  |  |  |  |

Inactivation natural molds and yeast data set at 250 MPa

Mean of replicas of two repetitions (log cfu/mL)

| Treatment time (min) | Repetition 1 | Repetition 2 |
| --- | --- | --- |
| 0,0 | 7,822495 | 7,781755 |
| 0,0 |  |  |
| 2,5 | 7,513217 | 7,492760 |
| 2,5 |  |  |
| 5,0 | 7,501744 | 7,434569 |
| 5,0 |  |  |
| 7,0 | 7,587711 | 7,537189 |
| 7,0 |  |  |

Inactivation natural molds and yeast data set at 400 MPa

No survivors at that pression level were counted
