# Supplementary material for: Use of high hydrostatic pressure to inactivate natural contaminating microorganisms and inoculated E. coli O157:H7 on Hermetia illucens larvae
Source: PLoS One. 2018 Mar 22;13(3):e0194477. doi: 10.1371/journal.pone.0194477 (PMC5864016; doi:10.1371/journal.pone.0194477)
Supplement: S2 Dataset — (DOCX) [file pone.0194477.s002.docx]

**S2_Data set for *E coli* O157H7**

Inactivation data set for *E coli* O157H7 used on model fitting

Data corresponding to four repetitions per treatment time (Log cfu/ml)

| Treatment time (min) | 250 MPa | | | | 350 MPa | | | | 400MPa | | | |
| --- | --- | --- | --- | --- | --- | --- | --- | --- | --- | --- | --- | --- |
|  | Repetition 1 | Repetition 2 | Repetition 3 | Repetition 4 | Repetition 1 | Repetition 2 | Repetition 3 | Repetition 4 | Repetition 1 | Repetition 2 | Repetition 3 | Repetition 4 |
| 0,00 | 7,040000 | 7,140000 | 7,040000 | 7,180000 |  |  |  |  |  |  |  |  |
| 5,00 | 6,360000 | 6,360000 | 6,650000 | 6,820000 |  |  |  |  |  |  |  |  |
| 7,50 | 6,530000 | 6,510000 | 6,340000 | 6,260000 |  |  |  |  |  |  |  |  |
| 11,00 | 6,420000 | 6,490000 | 6,380000 | 6,700000 |  |  |  |  |  |  |  |  |
| 15,00 | 6,320000 | 6,430000 | 6,480000 | 6,400000 |  |  |  |  |  |  |  |  |
| 0,00 |  |  |  |  | 7,359836 | 7,607455 | 7,453318 | 7,453318 |  |  |  |  |
| 2,50 |  |  |  |  | 3,881303 | 4,414973 | 4,712650 | 4,728624 |  |  |  |  |
| 5,00 |  |  |  |  | 4,107775 | 4,130869 | 4,063834 | 4,409651 |  |  |  |  |
| 7,50 |  |  |  |  | 4,069113 | 3,830909 | 3,870909 | 4,070113 |  |  |  |  |
| 10,00 |  |  |  |  | 3,801303 | 3,881303 | 3,558709 | 3,775974 |  |  |  |  |
| 0,00 |  |  |  |  |  |  |  |  | 7,350000 | 7,560000 | 7,300000 | 7,560000 |
| 1,00 |  |  |  |  |  |  |  |  | 4,200000 | 4,500000 | 4,380000 | 4,600000 |
| 2,50 |  |  |  |  |  |  |  |  | 3,960000 | 2,760000 | 3,430000 | 3,190000 |
| 5,00 |  |  |  |  |  |  |  |  | 3,030000 | 2,060000 | 2,490000 | 2,720000 |
| 7,00 |  |  |  |  |  |  |  |  | 1,980000 | 1,880000 | 2,320000 | 2,350000 |
|  |  |  |  |  |  |  |  |  |  |  |  |  |
